# The role of teachers' emotional facial expressions on student perceptions and engagement for primary school students-an experimental investigation

**DOI:** 10.3389/fpsyg.2025.1613073

**Published:** 2025-08-05

**Authors:** Marius Marici, Iasmina Iosim, Cornelia Diana Marin

**Affiliations:** ^1^Faculty of Education Sciences, Ştefan cel Mare University, Suceava, Romania; ^2^Faculty of Management and Rural Tourism, University of Life Sciences “King Mihai I” From Timişoara, Timişoara, Romania

**Keywords:** emotional display, teacher's emotions, students' perceptions, experimental design, facial expressions

## Abstract

**Introduction:**

This study aimed to investigate the impact of teachers' facial emotional displays (joy, neutrality, anger, and fear) on students' perceptions, compliance, and motivation.

**Methods:**

Using an experimental design, data were collected from 121 students, aged 912, (M = 10.3, SD = 0.78), from schools in Suceava, Romania. Participants were exposed to vignettes featuring teachers displaying different emotional expressions, and they completed questionnaires to assess their responses.

**Results:**

The intergroup comparisons revealed that joy had the most significant positive impact on students' willingness to participate, respect for the teacher, and overall enjoyment, while fear and anger led to negative outcomes.

**Discussion:**

The findings encourage the positive emotional expressions in fostering better student-teacher relationships and improving classroom engagement.

## 1 Introduction

The purpose of the present study was to investigate the effect of facial emotions of teachers on children's perceptions in school setting. The present advances in scientific research has already underlined the role of emotions in learning and cognitions (Pekrun, 2006), motivation (Linnenbrink-Garcia and Pekrun, 2011), social relationships (Meyer and Turner, 2006), behavior and discipline (Frenzel et al., 2009) wellbeing and mental health (Jennings and Greenberg, 2009) or even compliance (Meyer and Turner, 2006).

Theories also sustain the effect of emotions on school based activities. Broaden-and-Build Theory postulated by Fredrickson (2004), describes positive emotional states as a facilitator of aquiring or remembering new ideas or performing new behaviors (Rodrigo-Ruiz, 2016), unlike negative emotions. The Control-Value Theory asserts that motivation and attention increase with positive emotions, whereas negative emotions deplete focus and cognitive potential, hindering the learning process (Pekrun, 2006). Positive emotions improve concentration and simultaneously reduce the negative effects on learning outcomes (Salovey and Mayer, 1990). Emotions help reach deeper, more analytical thought processing, while negative ones act just in the opposite way (Evans and Stanovich, 2013).

The study was based on the observations of local debates in Suceava, concerning the fact that facial expressions are non-important issues in education and other elements such as teacher-student relationship or the clarity of explanations during classes matters the most. Facial expressions theme in school curriculum is really scarce and it is considered a negligible variable. In fact, some studies suggest that non-verbal clues may have a weaker effect (Thweatt and McCroskey, 1998). The present study focuses on facial expressions and not on other non-verbal means of communication because face is a top indicator of emotions, dispositions and attitude: “human faces are the most biologically significant visual stimuli. Faces contain a wealth of emotional cues” (Gu et al., 2023, p. 2).

Although the study focuses on the effect of four facial emotions on six themes or categories of effects, there is very limited research exactly on the topic, most indices regarding the associations between the variables being form nearby scientific fields. Studies focusing specifically on the facial expressions of teachers and their impact on children's perceptions in school settings are relatively limited, but growing. Most research on teacher emotions tends to focus broadly on emotional regulation, classroom management, and teacher-student relationships rather than isolating facial expressions as a specific variable (Sutton and Wheatley, 2003). However, studies that do investigate facial expressions often integrate them as part of a broader investigation into non-verbal communication, emotional climate, and their effects on student behavior and engagement (Hargreaves, 2000; Jennings and Greenberg, 2009; Pratama and Mukhlis, 2024). Children's perceptions are often examined within the context of teacher behaviors, including emotional displays, but these studies do not always isolate facial expressions from other factors like tone of voice or gestures (Meyer and Turner, 2006).

This study responds to the lack of experimental research focused solely on teachers' facial expressions, a core yet often overlooked element of classroom interaction. By contrasting four basic emotions in a child sample, it offers rare insight into how children interpret and react to distinct emotional cues, addressing a clear gap in both educational and developmental psychology.

## 2 Emotional dynamics and students' outcomes

### 2.1 Student's compliance to the teacher

When teachers exhibit positive emotions, such as enthusiasm, warmth, and encouragement, they create a supportive learning environment. This context enhances students' willingness to comply with instructions and classroom norms (Meyer and Turner, 2006). Positive display of emotions—smiling and keeping calm—help in the development of trust and rapport, which in turn allows the student to be more compliant with following directions and sets up an atmosphere of more willingness to participate (Jennings and Greenberg, 2009). Negative emotions, such as anger and frustration, may make students resistant, further leading to misbehavior and disruptions since it becomes a stressful and confrontational atmosphere (Sutton, 2004).

Facial expressions of teachers, such as smiling, frowning, and raising eyebrows, are non-verbal behaviors decoded by students—that can help or hinder students in the learning process (Ekman, 2003). For example, teachers who smile can make a classroom a positive venue for students, who then are more willing to learn, participate, and become more comfortable with the material they are learning (Pekrun and Linnenbrink-Garcia, 2014). On the other hand, negative facial expressions—frowning or looking angry—are likely to increase student anxiety and decrease motivation, creating a sense of unease that may obstruct learning in total (Hargreaves, 2000). As confirmed by research, students whose teachers are viewed as emotionally supportive through positive facial expressions are themselves more likely to develop positive attitudes toward learning and achieve more academically (Jennings and Greenberg, 2009).

The expression of joy functions not only as a social signal but also as an emotional regulator within the learning environment. According to Emotional Contagion Theory (Hatfield et al., 1993), students tend to unconsciously mimic the emotions displayed by teachers, meaning that a smiling teacher can induce a more cooperative and receptive emotional state. In parallel, Broaden-and-Build Theory Fredrickson's (2004) explains that positive emotions, such as joy, broaden students' thought-action repertoires, making them more open to complying with requests and engaging with classroom expectations without resistance.

### 2.2 Students' respect for the teacher

Displaying warmth and positivity in the classroom creates a respectful environment for students. When teachers show these emotions, students are more likely to reciprocate and show respect in return. This directly impacts the dynamics of the classroom. Smiling faces signal to students that their teachers are approachable and encouraging, leading students to participate respectfully in interactions (Jennings and Greenberg, 2009). This effect is reinforced by neurological evidence, which shows that positive emotional cues activate social bonding mechanisms, enhancing student trust and cooperation (Roorda et al., 2011; Lieberwirth and Wang, 2014).

In contrast, displays of anger from teachers lead to compliance driven by fear rather than genuine respect, as such expressions trigger stress responses in students, indicated by elevated cortisol levels (Sutton and Wheatley, 2003). Neutral expressions, while minimizing the risk of negative emotional impacts, generally result in flat responses from students, as they neither build trust nor promote engagement (Becker et al., 2014). Sadness, when expressed by teachers, has mixed outcomes; some students might empathize, but others may view it as a sign of weakness, undermining authority and respect (Hagenauer and Volet, 2014).

Respect is shaped not just by authority but also by emotional cues interpreted through a social lens. The Social Appraisal Theory (Manstead and Fischer, 2001) suggests that individuals interpret others' emotional expressions to assess social intentions and character. When teachers express joy, students are likely to appraise them as confident, emotionally stable, and supportive—traits associated with moral and intellectual authority. In contrast, anger or fear can be appraised as signs of volatility or weakness, undermining authentic respect.

### 2.3 Students' liking of the teacher's presence

Facial emotional expressions significantly influence students' attitudes toward their teacher's presence, shaping how much they like the teachers. Positive expressions, specifically joy (e.g., consistent smiling and nodding), create a perception of availability and openness. Frenzel et al. (2009) emphasize that such micro-expressions make students feel emotionally supported and increasing their appreciation for the teacher's presence.

In contrast, expressions of anger, such as frowning or glaring, have immediate negative effects (Marici et al., 2024). Certain facial expressions can trigger stress responses in students, leading to increased cortisol levels and feelings of discomfort (Sutton and Wheatley, 2003). This can make students less likely to enjoy being around the teacher and may associate the teacher with fear. Teachers who display neutral expressions, showing no emotion, may be perceived as uninterested, causing students to distance themselves. This lack of emotional connection can lead to a neutral or indifferent reaction toward the teacher. On the other hand, conveying sadness through subtle signals like drooping eyebrows or a downturned mouth can have mixed results. Students may feel sympathetic, fostering closeness, or may view it as a sign of weakness. This perception can undermine students' comfort with the teacher (Hagenauer and Volet, 2014).

Facial joy has a relational function—it facilitates emotional closeness and likability. According to Keltner and Haidt (1999), positive emotional displays such as smiling help maintain social bonds and signal affiliative intent. A teacher who frequently smiles is perceived as emotionally available, which fosters comfort and personal connection.

### 2.4 Willingness to work harder

Positive expressions make students feel valued and thus enhance their motivation (Frenzel et al., 2009). The Emotional Contagion Theory claims that students often mirror their teacher's positive emotions, setting up a mutual environment inside the classroom (Hatfield et al., 1993). These further act as social rewards, reinforcing effort and participation from the students by increasing their intrinsic motivation to do the tasks (Pekrun, 2006). Neutral expressions involve minimal face movements and emotional displays, which do not necessarily lead to negative responses but basically kill the interaction. As such, students may sense the teacher as emotionally removed. Consequently, they disconnect and show indifference because they obtain no signs for welcome or, at the very least, display of concern (Becker et al., 2014). Students connect their interest in a subject to the teacher's positive emotional approach, enhancing their intrinsic motivation to work (Frenzel et al., 2021).

Anger, expressed by frowning and glaring or other aggressive facial cues, can enforce immediate compliance but often elicits discomfort and stress responses in students (Sutton and Wheatley, 2003). Facial expressions provide an increase in cortisol levels among students, and students may experience more fear rather than motivation. Again, this was supported by the Social Appraisal Theory (Manstead and Fischer, 2001), whereby the students construe angry displays as hostile, which would hurt their relationship with the teacher and also decrease overall motivation. Students may feel disturbed by fearful expressions: when the teacher acts fearfully, students may construe this display as one of lack of control/authority, which may diminish their sense of confidence in the teacher's guidance. Negative utterances, on the part of the teacher, may lead to doubt, distress, or low participation as well because students would regard it as a classroom environment that is somehow unstable or unsupportive (Hagenauer and Volet, 2014).

### 2.5 Child attentiveness and teachers' facial expressions

Positive emotional displays, such as smiling, make the classroom a safe place and raise students' moments of focus (Pianta et al., 2008). Frenzel et al. (2009) also showed that smiles are an expression of joy that can create an emotional contagion effect, heightening students' alertness and involvement. Owens et al. (2018) further supported this by stating that smiling increases the likelihood of cognitive engagement, thus helping students to stay on task. Consistent displays of positivity among instructors (Pallini et al., 2019) help in trying to modulate students' attention and conduct and reduce distracting behavior. These kinds of affective behaviors create an environment that is ripe for learning—a setting wherein students are interested in being attentive (Grove, 2019).

Facial joy fosters a secure classroom climate that supports student attention. According to Broaden-and-Build Theory Fredrickson's (2004), positive emotions like smiling expand cognitive focus and signal safety. When teachers appear emotionally engaged, students feel more at ease and become more attentive and mentally involved.

### 2.6 Teacher's facial emotions and perceptions of teacher's personality features

A teacher who smiles a lot is typically viewed as easy to approach, empathetic, and trustworthy—those being key features of a supportive teacher (Kerssen-Griep et al., 2003). Positive facial expressions, such as smiles, also lead to perceptions of openness and enthusiasm, characteristics of a teacher who is encouraging and friendly in nature (Wang et al., 2017). In contrast, negative facial expressions can include frowns and/or the display of frustration, which often result in teachers being perceived as more authoritarian and unapproachable in nature. Correspondingly, teachers with these expressions may be perceived as strict, controlling, or unsupportive (Becker et al., 2014). These emotional displays can make a teacher appear more dominating or harsh, and it reduces the relational warmth between teacher and student (Hamre and Pianta, 2006).

Students infer personality traits from emotional expressions, often subconsciously. Research grounded research (Asch, 1946) suggests that warmth and expressiveness heavily influence how individuals judge others' dispositions. Smiling teachers are typically perceived as competent, kind, and trustworthy—attributes associated with effective teaching. In contrast, negative expressions may trigger impressions of authoritarianism or disconnection, distancing students both emotionally and cognitively.

## 3 The present research

The present research aims to examine how emotions influence children's perceptions of their teachers, specifically investigating whether there is a difference between positive and negative emotions expressed by teachers during lessons. Additionally, the study explores how children perceive certain teacher competencies based on the emotions conveyed. The study is a quantitative one, having an experimental design, based on questionnaires, filled up in the classroom. The data collected were then analyzed to address the core research question: How do facial emotions change children's perceptions?

This study brings both theoretical and practical contributions by emphasizing the overlooked yet powerful role of teachers' facial emotional expressions in shaping student engagement and perception. While salespeople and marketing professionals are routinely trained to smile and use positive facial cues to influence others, teachers—despite their constant interpersonal contact—are rarely guided to harness these non-verbal tools. The study fills this gap in research addressing practical didactic behaviors.

## 4 Research methodology

### 4.1 Instruments

#### 4.1.1 Vignette creation

**Figure 1:**
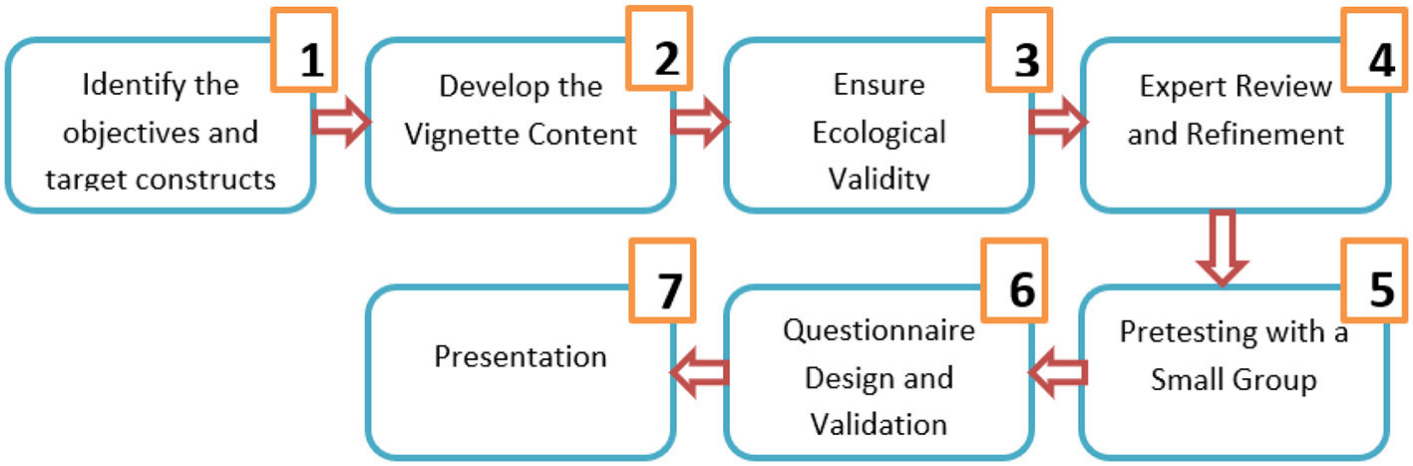
The measurement instrument development flowchart.

The experimental design required the creation of a vignette. The creation process, including all the steps, is described below (see Figure 1).

1. *Identify the Objective and Target Constructs*: The vignette aimed to measure students' willingness to complete additional exercises, evaluate the teacher's personality traits, motivation to listen, respect, and liking for the teacher. The constructs under investigation (willingness, respect, motivation, etc.) were clearly defined, what should be and what should not be included.
2. *Develop the Vignette Content*: A realistic classroom scenario was developed to reflect the teacher-student interaction. The setting of a Romanian language lesson was chosen to ensure familiarity with the context. The vignette focused on a specific instructional event: the teacher giving an additional worksheet and specific instructions. The scenario was designed to elicit genuine reactions regarding teacher authority and student motivation. The situation was selected as a result of multiple session discussions with students from university.
3. *Ensure Ecological Validity*: To ensure the scenario resembled real-life situations students might encounter, the vignette was kept simple and direct. The teacher's role and actions in the vignette (giving a worksheet after a long lesson) were plausible and commonly observed in educational settings. More students were involved and they evaluated how much the scenario reflects reality and suggested changes. This happened more times until the scenario felt fit for research.
4. *Expert Review and Refinement*: The vignette was reviewed by a group of educational psychologists and teachers to ensure the realism and clarity of the scenario. They provided feedback on whether the vignette effectively represented teacher-student dynamics and whether the instructions were clear and unbiased. Based on their input, minor revisions were made to the phrasing of the teacher's instructions to avoid leading the participants to specific responses. For example, words were replaced, or were added extra information.
5. *Pretesting with a Small Group*: The vignette was pilot-tested with a small group of students to assess its comprehensibility and to identify any confusing aspects. The pilot participants were also asked if they understood the task and if they felt the situation was believable. Feedback indicated that the vignette was well-understood, and no major revisions were required.

To determine if children correctly identify emotions based on human facial expressions, a pre-test was conducted with 30 participants. In this test, the children were asked to label each face with the corresponding emotion. The questionnaires and pre-test were administered during regular class hours under the joint supervision of both myself and the teacher. The children filled out the forms manually on A4 sheets after receiving all necessary instructions. No personal information was required, and the participants were informed that they could withdraw from the study at any time if they chose to do so.

“Please indicate what do these human faces express. Write in the table the corresponding letter.”

**A. B. C. D**. (faces were not published here owing to the copyright policy).

All 30 respondents, participating in the pilot study, indicated the right emotion, which indicated a good fit for our research.

6. *Questionnaire Design and Validation*: Along with the vignette, a Likert scale was used for responses to the questions. The questions were reviewed by experts to ensure they appropriately captured the intended constructs. The questions were also piloted with the same small group of students, confirming that the scales were clear and relevant.
7. *Presentation*: The vignette was presented in a consistent format to all participants in the group to avoid any presentation bias. After refining the vignette and pretesting, the final version was administered in a larger study. The vignette and the questions are presented below:

“Imagine that the lady in the picture is your teacher. You have just finished a Romanian language lesson with her, where you learned about nouns. Because the lesson was long, the teacher gives you an additional worksheet to deepen your understanding and says, “Please complete exercises 1, 3, and 4 now.” As a response to this situation students were asked to answer the following questions: “(1) How willing would you be to complete the exercises requested now? (2) How much do you respect the teacher in the photo? (3) How much do you enjoy her company as a person? (4) How willing would you be to do some additional exercises at home? (5) If the teacher in the photo were your teacher, how motivated would you be to listen to her attentively in class? (6) Please indicate how much of these features does your teacher have?”

The selection of a female facial model was intentional, reflecting the demographic reality that the majority of primary school teachers are women. This choice enhances ecological validity by aligning the experimental stimuli with students' typical classroom experience. However, the potential for gender-related perceptual bias remains relevant and should be acknowledged, as students may respond differently to emotional expressions depending on the perceived gender of the teacher.

9. *Methodological contribution of the chosen experimental design*. The experimental design using standardized emotional stimuli and vignettes ensures high internal validity by isolating facial expressions as the only manipulated variable. This allows for causal inferences about their specific impact on student perceptions. Additionally, applying this controlled method with a child sample adds methodological value to a field dominated by self-report or observational studies.

Static images allow for controlled isolation of facial expressions, reducing confounding variables present in live interactions. While they don't fully capture classroom dynamics, they reliably trigger emotional appraisals, offering valid initial insights. Still, results should be interpreted with caution regarding generalizability to real-time teaching contexts.

#### 4.1.2 Measures

The students evaluated their teacher's personality, motivation to listen carefully, how much they respect their teacher, how much they like her presence, how willing they would be to do some additional exercises for homework?

*Karolinska Directed Emotional Faces (KDEF)*—The KDEF, developed by Lundqvist and colleagues in 1998, includes 4,900 images depicting the faces of 70 individuals expressing seven basic human emotions, from five different angles. Of the 70 participants, 35 are male and 35 female, with males having no beard, mustache, or glasses, and females devoid of earrings, glasses, or makeup (Lundqvist et al., 1998). For this study, we selected images of one female displaying four different emotions from a single frontal angle. The images were printed in color on an A4 sheet for clear visibility. The images were adapted by us based on the original. We removed the colored background and lighten the faces more so the facial indices be observed better. The present study selected only 4 emotional faces: happiness, sadness, fear or neutrality. They rather correspond to the most common situation in classroom. These emotions are commonly chosen due to their universal recognition and impact on children's behavior other studies using them too (Mastorogianni et al., 2024). Happiness and sadness are basic emotions that children can easily identify and are significant in understanding social interactions and responses in an educational setting. Fear is relevant as it often triggers compliance behaviors due to its association with avoidance and authority, while neutrality serves as a baseline for comparison against other emotional states. This instrument was used in previous research and demonstrated good reliability and fidelity (Lin et al., 2024). Neutrality was included as one of the four emotional facial expressions presented through images, alongside joy, fear, and anger. Although neutrality is not an emotion in the classical sense, it is commonly used in experimental designs as a baseline or control condition due to its lack of expressive content. This allows researchers to compare participants' responses to emotionally expressive faces against a neutral facial display, helping isolate the specific effects of emotional valence and arousal.

*Questions assessing teacher-student interactions*—Since no existing tool fully met the needs of our research, we developed a new instrument. After several discussions and revisions, we finalized a questionnaire that included a hypothetical classroom scenario (a vignette) followed by four teacher-student relationship related questions. In this scenario, the children were asked to imagine completing a Romanian language class where the teacher gave them additional exercises after a long lesson. The questions assessed how willing they would be to complete the exercises, respect the teacher, enjoy her company, and whether they would voluntarily do extra homework. An additional question was posed to gauge their motivation to listen attentively to the teacher in class. The responses were rated on a 10-point Likert scale, where 0 indicated “not at all” and 9 indicated “very much”.

The validation process of the instruments involved both content and construct validation steps to ensure measurement accuracy and relevance. Content validity was established through expert review by educational psychologists and teachers, who assessed the clarity, realism, and alignment of the vignette and questionnaire items with the targeted constructs (e.g., compliance, respect, motivation). Construct validity was supported through pilot testing with a small student group, confirming that the scales accurately captured the intended psychological dimensions. The use of a 10-point Likert scale provided sufficient variability and sensitivity in responses, suitable for children familiar with school-based rating formats. Demographic variables—such as gender, age, family structure, and rural/urban background—were included based on prior research indicating their moderating role in emotional interpretation and teacher-student dynamics, thereby allowing for richer contextual analysis.

*Rokeach Value Survey (RVS)*—Additionally, the Rokeach Value Survey (RVS), authored by Milton Rokeach in 1973, was used to explore students' perceptions of the teacher's personality. The instrument includes two subscales—terminal and instrumental values. For our study, we focused on the instrumental values subscale, which contains 18 personality traits (such as ambition, honesty, kindness, and responsibility), and the students rated them on an 11-point Likert scale, where 0 indicated “not at all” and 11 indicated “very much”. The “Cheerful” personality trait was eliminated from our study as it referred directly to the smiling face image, and there was no reason to ask respondents to indicate how cheerful they perceive their smiling teacher. The instrument was used before in other studies and showed good reliability (Marici et al., 2023).

The demographic variables used were: age, sex, rural or urban background, marital status of parents, number of children in family, parents' income, working abroad or at home or how well they learn at school and they were used for the description of the participants.

### 4.2 Participants

This study involved a sample of 121 students from third and fourth grades, who were randomly divided into four groups (one control group and three experimental groups). The participants were from Suceava, Romania, and were children in the 3rd, 4th, 5th, or 6th grade (see Table 1).

**Table 1:** Key features of participants in the study.

**Variables**	**Levels**	**Characteristics**
Age	14.9% were 9 years old, 49.6% were 10, 29.8% were 11, 5.8% were 12 years old.	*M =* 10.3, SD = 0.78 Min 9, Max 12
Sex	55.4% females and 44.6% males	
Background	Urban environment (73.6%) Rural areas 26.4%	
Status	79.3% married 6.6% separated 8.3% divorced 3.3% one parent deceased 2.5% living together but not married	
Children	14% one child 54.5% two children 18.2% three children 6.6% four children 6.7% more than four children	*M =* 2.47, SD = 1.42 Min 1, Max 12
Income	11.6% Less than we need 47.9% Exactly as we need 28.9% More than we need 11.6% Much more than we need	
Work	61.7% Both work in the country 26.7% One parent works abroad 4.2% Both parents work abroad 7.5% None work	
How well they learn at school?		*M =* 7.47, SD = 1.52 Min 1, Max 9

*Access to participants*: The participants were accessed in public school setting. The researcher had two assistants who went to schools, spoke with principals, and collected data. The data was collected during regular classes with the assistance of the teachers and research assistants.

*Selection criteria*: We collected the data from pupils who were in the 3rd, 4th, or 5th grade.

In this study, the 121 students were randomly assigned into four groups: Joy (*n* = 29), Anger (*n* = 29), Neutrality (*n* = 28), and Fear (*n* = 30). Inclusion criteria required students to be enrolled in grades 3–5 and to have obtained parental consent. Exclusion criteria included visual impairments, physical or cognitive difficulties that could interfere with recognizing facial expressions. Randomization was conducted at the classroom level using a simple random assignment process to ensure group equivalence and reduce selection bias.

Although the sample includes a detailed demographic breakdown, the study should reflect more critically on its representativeness. The high proportion of urban participants (73.6%) and families reporting average or above-average income may limit the generalizability of results to more socioeconomically diverse or rural populations. Additionally, the reliance on students from a single geographic area may introduce regional cultural biases in emotional interpretation, suggesting the need for broader sampling in future research to enhance external validity.

The study used random assignment to allocate participants to one of the four emotional conditions, ensuring that group differences were not due to pre-existing characteristics.

### 4.3 Collecting data

The data for this study were collected from students attending several schools in Suceava, Romania. We spoke in advance with the school principals, who facilitated our access to the children. The experiment took place during regular school hours to maintain a familiar environment for the students, minimizing any external influences on their responses.

For the experiment, we used a controlled design where students were divided into groups, with each group being exposed to a teacher's facial emotional display—joy, neutrality, anger, or fear. Students were asked to imagine a teacher giving them additional tasks after a Romanian language lesson. This vignette served as the foundation for evaluating how the teacher's emotional expression affected the students' perceptions of several issues. After the vignette was presented, students completed a questionnaire using a Likert scale to rate their responses to different questions about the teacher or about them. Data was collected on paper and then introduced in software.

### 4.4 Ethics

The participation to the pretest and the experiment itself involved human participants who voluntarily took part. Before the experiment, all participants gave their informed consent in writing, ensuring full awareness of the procedure. Only anonymized data were processed to protect participant privacy. The study adhered to the ethical guidelines established by the Declaration of Helsinki, and the procedures for data collection and the validation experiment were approved by the ethics board of the institution associated with the first author (MM).

The study ensured ethical controls by obtaining parental consent and child assent prior to participation. Procedures were designed to minimize psychological risk, using only brief, age-appropriate stimuli and ensuring the presence of supervising adults throughout the experiment.

### 4.5 Hypotheses

The study has 5 hypotheses which are formulated below:

***H1***: Students' compliance rate will be higher when teachers smile as compared to the other emotions.
***H2:*** Students will respect more the smiling teacher than the teachers displaying other emotions.
***H3:*** Students will like more the company of the smiling teacher, than those displaying other emotions.
***H4:*** Students will be more willing to do some extra exercises in the situation of the smiling teacher as compared to the other emotions displayed.
***H5:*** Students will listen to the student more attentively when teacher displays a smiling face than other emotions.
***H6:*** Smiling faces will be better associated with teacher's positive personality features, than other emotions.

The present study brings novelty by isolating teachers' facial expressions as the sole independent variable, a rarely explored factor in previous research where emotional impact is often confounded with voice, gestures, or general demeanor. Unlike earlier studies, this research systematically examines six distinct student responses—ranging from compliance and motivation to personality attribution—offering a multifaceted view of emotional influence in education. Particularly innovative is the inclusion of perceived personality traits (H6), connecting emotional display to character judgments, not just classroom behavior. Using an experimental vignette design with standardized emotional stimuli among children aged 9–12, the study adds methodological rigor and fills a gap in empirical data from Eastern European contexts, which remain underrepresented in international literature.

### 4.6 Statistical procedure

To analyze the data, we used One-Way ANOVA to examine the impact of the teacher's emotional displays (joy, neutrality, anger, and fear) on various dependent variables, including students' compliance, respect, motivation to listen, enjoyment of the teacher's presence or teacher's personality. The ANOVA allowed us to determine whether there were significant differences between the groups based on the teacher's emotional expression. When the ANOVA indicated significant differences, we followed up with *post-hoc* tests, such as Fisher's or Welch's tests, to pinpoint the specific group comparisons (e.g., joy vs. neutrality) that were driving these effects. In addition, we used descriptive statistics and the software used was Jamovi. The software is an open-source statistical software designed for data analysis, offering an intuitive interface and comprehensive tools for conducting statistical tests, visualizations, and data manipulation. It is widely used in research and education due to its user-friendly design and integration of advanced features like R syntax (jamovi). Participants were supervised by trained assistants to ensure standardized procedures. No missing data were reported; listwise deletion would apply if needed. Jamovi was chosen for its user-friendly interface and familiarity with it.

## 5 Results

### 5.1 Descriptive statistics

The descriptive analysis indicated that the responses varied according to exposure to facial expressions and the means and the standard deviations for the total score formed from all answers are presented below (Table 2).

**Table 2:** Descriptive statistics by facial expression.

**Descriptive analysis**	**Joy**	**Anger**	**Neutrality**	**Fear**
Mean	8.96	5.57	5.53	4.62
Standard deviation	0.84	2.47	1.32	1.60
*N*	29	29	28	30

### 5.2 Hypotheses testing

In order to test the 6 hypotheses, we performed The One-Way ANOVA analysis which indicated the following significant differences between students' responses according to teachers' emotional displays. The non-significant results are not presented in the Table 3.

**Table 3:** Results for ANOVA (Welch) and *post-hoc* comparisons.

**Feature ANOVA Welch [F_(3, df2)_, *p*]**	**Comparisons *post-hoc* [*M* (SD); *t*_(df)_, *p*]**
**1. Ambitious** *F*_(3, 64.6)_ = 29.869, *p* < 0.001	Joy vs. Neutrality: 8.56(2.38) vs. 2.73(2.79); *t*_(56.6)_ = −8.70, p <0.001, *d =* 2.25 Joy vs. Anger: 8.56(2.38) vs. 5.96(3.32); *t*_(52.6)_ = 3.48, *p* = 0.005, *d* = 0.9 Joy vs. Fear: 8.56(2.38) vs. 3.71(2.88); *t*_(57.6)_ = 7.17, *p* = 0.004, *d =* 1.84 Anger vs. Neutrality: 5.96(3.32) vs. 2.73(2.79); *t*_(117)_ = −4.37, *p* < 0.001, *d =* −1.05 Anger vs. Fear: 5.96(3.32) vs. 3.71(2.88); *t*_(57.3)_ = 2.83, *p* = 0.032, *d =* 0.72
**2. Broadminded** *F*_(3, 62.6)_ = 42.599, *p <* 0.001	Joy vs. Neutrality: 8.83(1.76) vs. 3.63(3.13); *t*_(45.7)_ = −7.92, *p <* 0.001, *d =* 2.05 Joy vs. Anger: 8.83(1.76) vs. 5.43(3.58); *t*_(42.2)_ = 4.66, *p <* 0.001, *d =* 1.21 Joy vs. Fear: 8.83(1.76) vs. 3.19(2.57); *t*_(53.2)_ = 10.01, *p <* 0.001, *d =* 2.56 Anger vs. Fear: 5.43(3.58) vs. 3.19(2.57); *t*_(52.5)_ = 2.79, *p <* 0.035, *d =* 0.72
3. **Capable** *F*_(3, 52.9)_ = 58.998, *p <* 0.001	Joy vs. Neutrality: 9.56(0.67) vs. 5.20(2.97); *t*_(32)_ = −7.84, *p <* 0.001, *d =* 0.72 Joy vs. Anger: 9.56(0.67) vs. 5.56(3.69); *t*_(31)_ = 5.83, *p <* 0.001, *d =* 1.51 Joy vs. Fear: 9.56(0.67) vs. 4.25(2.85); *t*_(33.5)_ = 10.07, *p <* 0.001, *d =* 2.56
4. **Clean** *F*_(3, 57.6)_ = 35.700, *p <* 0.001	Joy vs. Neutrality: 9.50(0.97) vs. 5.83(2.27); t_(39.3)_ = −8.11, *p <* 0.001, *d =* 2.1 Joy vs. Anger: 9.50(0.97) vs. 6.33(3.48); *t*_(33.5)_ = 4.79, *p <* 0.001, *d =* 1.24 Joy vs. Fear: 9.50(0.97) vs. 6.03(2.73); *t*_(37.7)_ = 6.63, *p <* 0.001, *d =* 1.69
5. **Courageous** *F*_(3, 63.7)_ = 46.812, *p <* 0.001	Joy vs. Neutrality: 8.41(2.41) vs. 4.33(2.83); *t*_(56.1)_ = −5.96, *p <* 0.001, *d =* 1.55 Neutrality vs. Anger: 4.33(2.83) vs. 6.70(3.28); *t*_(56.8)_ = −2.99, *p =* 0.021, *d =* 0.78 Neutrality vs. Fear: 4.33(2.83) vs. 1.54(2.20); *t*_(54.8)_ = 4.28, *p <* 0.001, *d =* 1.1 Joy vs. Fear: 8.41(2.41) vs. 1.54(2.20); *t*_(56.6)_ = 11.48, *p <* 0.001, *d =* 2.98 Anger vs. Fear: 6.70(3.28) vs. 1.54(2.20); *t*_(50.5)_ = 7.17, *p <* 0.001, *d* = 1,85
6. **Forgiving** *F*_(3, 58.6)_ = 40.469, *p <* 0.001	Joy vs. Neutrality: 9.41(1.24) vs. 4.80(3.19); *t*_(37.8)_ = −7.35, *p <* 0.001, *d =* 1.83 Joy vs. Anger: 9.41(1.24) vs. 3.83(3.62); *t*_(35.9)_ = 7.97, *p <* 0.001, *d =* 1.95 Joy vs. Fear: 9.41(1.24) vs. 5.90(2.85); *t*_(41.5)_=6.24, *p <* 0.001, *d =* 1.44
7. **Helpful** *F*_(3, 59.2)_ = 49.574, *p <* 0.001	Joy vs. Neutrality: 9.26(1.33) vs. 4.33(3.12); *t*_(39.3)_ = −7.97, *p <* 0.001, *d =* 1.97 Joy vs. Anger: 9.26(1.33) vs. 4.53(3.83); *t*_(35.9)_ = 6.37, *p <* 0.001, *d =* 1.61 Joy vs. Fear: 9.26(1.33) vs. 3.77(2.90); *t*_(42.5)_ = 9.53, *p <* 0.001, *d =* 2.06
8. **Honest** F_(3, 61.2)_ = 27.568, *p <* 0.001	Joy vs. Neutrality: 9.13(1.27) vs. 6.23(2.41); *t*_(44.1)_ = −5.81, *p <* 0.001, *d =* 1.48 Joy vs. Anger: 9.13(1.27) vs. 6.23(3.20); *t*_(38)_ = 4.61, *p <* 0.001, *d =* 1.17 Joy vs. Fear: 9.13(1.27) vs. 5.41(2.33); *t*_(46.8)_ = 7.74, *p <* 0.001, *d =* 1.80
9. **Imaginative** *F*_(3, 56.9)_ = 47.022, *p <* 0.001	Joy vs. Neutrality: 9.53(1.10) vs. 4.40(3.18); *t*_(35.9)_ = −8.35, *p <* 0.001, *d =* 2.02 Joy vs. Anger: 9.53(1.10) vs. 5.73(3.80); *t*_(33.9)_ = 5.25, *p <* 0.001, *d =* 1.32 Joy vs. Fear: 9.53(1.10) vs. 4.51(3.03); *t*_(38.1)_ = 8.63, *p <* 0.001, *d =* 2.00
10. **Independent** *F*_(3, 63)_ = 27.092, *p <* 0.001	Neutrality vs. Fear: 7.60(1.40) vs. 3.71(2.54); *t*_(47)_ = 7.42, *p <* 0.001, *d =* 1.82 Joy vs. Anger: 8.83(1.89) vs. 7.00(2.77); *t*_(51.2)_ = 2.99, *p* = 0.022, *d =* 0.77 Joy vs. Fear: 8.83(1.89) vs. 3.71(2.54); *t*_(54.4)_ = 8.94, *p <* 0.001, *d =* 2.33 Anger vs. Fear: 7.00(2.77) vs. 3.71(2.54); *t*_(58.2)_ = 4.82, *p <* 0.001, *d =* 1.26
11. **Intellectual** *F*_(3, 61.6)_ = 14.974, *p <* 0.001	Joy vs. Neutrality: 9.60(1.38) vs. 7.93(1.71); *t*_(53.8)_ = −4.12, *p =* 0.001, *d =* 1.06 Joy vs. Anger: 9.60(1.38) vs. 7.24(3.38); *t*_(36.8)_ = 3.48, *p <* 0.001, *d =* 0.91 Joy vs. Fear: 9.60(1.38) vs. 6.77(2.15); *t*_(51.3)_ = 6.11, *p <* 0.001, *d =* 1.52
12. **Logical** *F*_(3, 59.9)_ = 40.134, *p <* 0.001	Joy vs. Neutrality: 9.40(1.22) vs. 6.20(1.82); *t*_(48.7)_ = −7.89, *p <* 0.001, *d =* 2.01 Joy vs. Anger: 9.40(1.22) vs. 6.30(3.65); *t*_(35.4)_ = 4.40, *p <* 0.001, *d =* 1.11 Joy vs. Fear: 9.40(1.22) vs. 4.63(2.60); *t*_(41.1)_ = 9.07, *p <* 0.001, *d =* 2.20
13. **Loving** *F*_(3, 58.2)_ = 71.414, *p <* 0.001	Joy vs. Neutrality: 9.80(1.09) vs. 4.60(2.45); *t*_(40.1)_ = −10.6, *p <* 0.001, *d =* 2.61 Joy vs. Anger: 9.80(1.09) vs. 3.43(3.71); *t*_(34)_ = 9.01, *p <* 0.001, *d =* 2.26 Joy vs. Fear: 9.80(1.09) vs. 4.87(2.88); *t*_(38.7)_ = 8.87, *p <* 0.001, *d =* 2.12
14. **Obedient** *F*_(3, 51.8)_ = 70.516, *p <* 0.001	Joy vs. Neutrality: 9.73(0.58) vs. 4.73(2.93); *t*_(31.3)_ = −9.15, *p <* 0.001, *d =* 2.49 Joy vs. Anger: 9.73(0.58) vs. 3.93(4.20); *t*_(30.1)_ = 7.47, *p <* 0.001, *d =* 1.89 Joy vs. Fear: 9.73(0.58) vs. 4.58(2.95); *t*_(32.4)_ = 9.52, *p <* 0.001, *d =* 2.45
15. **Polite** *F*_(3, 52.8)_ = 44.583, *p <* 0.001	Joy vs. Neutrality: 9.70(0.70) vs. 5.86(3.00); *t*_(32.2)_ = −6.81, *p <* 0.001, *d =* 1.76 Joy vs. Anger: 9.70(0.70) vs. 4.50(4.01); *t*_(30.8)_ = 6.99, *p <* 0.001, *d =* 1.80 Joy vs. Fear: 9.70(0.70) vs. 5.51(3.09); *t*_(33.2)_ = 7.33, *p <* 0.001, *d =* 1.71
16. **Responsible** *F*_(3, 63.7)_ = 17.923, *p <* 0.001	Joy vs. Neutrality: 9.16(1.87) vs. 6.46(2.14); *t*_(57)_ = −5.19, *p <* 0.001, *d =* 1.37 Joy vs. Anger: 9.16(1.87) vs. 5.46(3.80); *t*_(42.3)_ = 4.78, *p <* 0.001, *d =* 1.13 Joy vs. Fear: 9.16(1.87) vs. 5.61(2.52); *t*_(55.4)_ = 6.25, *p <* 0.001, *d =* 1.61
**17. Self-controlled** *F*_(3, 61.9)_ = 50.433, *p <* 0.001	Joy vs. Neutrality: 9.26(1.28) vs. 6.16(1.85); *t*_(57)_ = −5.19, *p <* 0.001, *d =* 1.88 Joy vs. Anger: 9.26(1.28) vs. 5.06(3.73); *t*_(42.3)_ = 4.78, *p <* 0.001, *d =* 1.39 Joy vs. Fear: 9.26(1.28) vs. 4.03(2.30); *t*_(55.4)_ = 6.25, *p <* 0.001, *d =* 2.22

*M* (SD) = mean (standard deviation); *t*-values are reported with df in parentheses. df = 117. Non-significant differences are not reported in the table.

***Hypothesis 1***: Students' *compliance* rate will be higher when teachers smile as compared to the other emotions.

The One-Way ANOVA showed a significant effect of emotional condition on participants' willingness to complete the requested exercises, *F*_(3, 64.5)_ = 7.66, *p* < 0.001. *Post-hoc* comparisons using Welch's test revealed that participants in the Joy condition (*M* = 7.60, SD = 1.69) expressed significantly greater willingness compared to those in the Neutrality condition (*M* = 6.03, SD = 1.73), *t*_(117)_ = −2.97, *p* = 0.019, *d* = 0.92. Additionally, the Joy condition also resulted in significantly higher willingness scores than the Fear condition (*M* = 5.26, SD = 2.36), *t*_(117)_ = 4.47, *p* < 0.001, *d* = 1.14. In the rest of the situations, we did not find any significant differences. The hypothesis is partially confirmed as two from three comparisons confirmed.

***Hypothesis 2**:* Students will *respect* more the smiling teacher than the teachers displaying other emotions.

The analyses indicated significant differences between groups: *F*_(3, 61)_ = 14.8, *p* < 0.001. Participants reported significantly higher scores in the Joy condition (*M* = 8.50, SD = 1.01) compared to Fear condition (*M* = 5.65, SD = 2.65), *t*_(38.7)_ = 5.59, *p* < 0.001, *d* = 1.41, compared to the Neutrality condition (*M* = 7.37, SD = 1.40), *t*_(52.7)_ = −3.59, *p* = 0.004, *d* = 0.93, or to Anger (*M* = 6.37, SD = 2.83), *t*_(36.2)_ = 3.88, *p* = 0.002, *d* = 1. The hypothesis is fully confirmed as three from three comparisons confirmed.

***Hypothesis 3**:* Students will *like* more the company of the smiling teacher, than those displaying other emotions.

The One-Way ANOVA (Welch's test) revealed a significant effect of emotional condition on participants' reported enjoyment of the person's company, *F*_(3, 63.8)_ = 34.8, *p* < 0.001. *Post-hoc* analyses indicated that participants in the Joy condition (*M* = 8.20, SD = 1.79) reported significantly greater enjoyment compared to those in the Neutrality condition (*M* = 4.90, SD = 2.47), *t*_(117)_ = −5.08, *p* < 0.001, *d* = 1.53, the Anger condition (*M* = 4.63, SD = 3.23), *t*_(117)_ = 5.49, *p* < 0.001, 1.37, and the Fear condition (*M* = 2.97, SD = 2.36), *t*_(117)_ = 8.13, *p* < 0.001, *d* = 2.49. Additionally, participants in the Neutrality condition reported significantly greater enjoyment compared to the Fear condition, *t*_(117)_ = 3.00, *p* = 0.017. The hypothesis is totally confirmed as it confirmed in three from three situations tested.

***Hypothesis 4:*** Students will be more *willing to do some extra exercises* in the situation of the smiling teacher as compared to the other emotions displayed.

A One-Way ANOVA revealed a significant effect of emotional condition on participants' willingness to do *additional exercises at home*, *F*_(3, 63.4)_ = 8.05, *p* = 0.001. *Post-hoc* tests (Fisher's LSD) showed that participants in the Joy condition (*M* = 7.23, SD = 1.61) reported significantly greater willingness compared to those in the Neutrality condition (*M* = 5.03, SD = 2.52), *t*_(117)_ = −3.54, *p* = 0.003, *d* = 1.04, and also compared to participants in the Fear condition (*M* = 5.03, SD = 2.69), *t*_(117)_ = 3.56, *p* = 0.003, *d* = 0.99. In the rest of the situations, we did not find any significant differences. The hypothesis is partially confirmed as two from the three conditions confirmed.

***Hypothesis 5**:* Students will listen to the student *more attentively* when teacher displays a smiling face than other emotions.

A One-Way ANOVA (Games-Howell test) indicated a significant effect of emotional condition on participants' motivation to attentively listen in class, *F*_(3, 61.7)_ = 23.9, *p* < 0.001. *Post-hoc* comparisons revealed that participants reported significantly higher motivation in the Joy condition (*M* = 8.33, SD = 1.35) compared to the Neutrality condition (*M* = 5.57, SD = 2.39), *t*_(45.8)_ = −5.53, *p* < 0.001, *d* = 1.43, the Fear condition (*M* = 4.48, SD = 2.49), *t*_(46.5)_ = 7.55, *p* < 0.001, *d* = 1.91, and the Anger condition (*M* = 6.37, SD = 3.08), *t*_(39.7)_ = 3.20, *p* = 0.014, *d* = 0.82. The hypothesis is totally confirmed as three from the three tested comparisons are significant.

***Hypothesis 6**:* Smiling faces will be better associated with *teacher's positive personality features*, than other emotions.

Below are the results for Hypothesis 6, which explored whether smiling faces would be more strongly linked to a teacher's positive personality features compared to other emotional expressions.

In examining 17 distinct traits, Joy was significantly different from all three comparison emotions (Neutrality, Anger, and Fear) in 15 of these traits, suggesting a robust and consistent pattern where Joy scores stood apart from the other conditions tested in hypotheses. In the remaining two traits (Courageous and Independent), Joy still differed significantly from two of the other emotions, but not all three. Overall, these findings underscore that Joy emerges as a uniquely influential emotional state for most attributes measured, with particularly pronounced contrasts when compared to Neutrality, Anger, and Fear. The hypothesis was partially confirmed.

**Figure 2:**
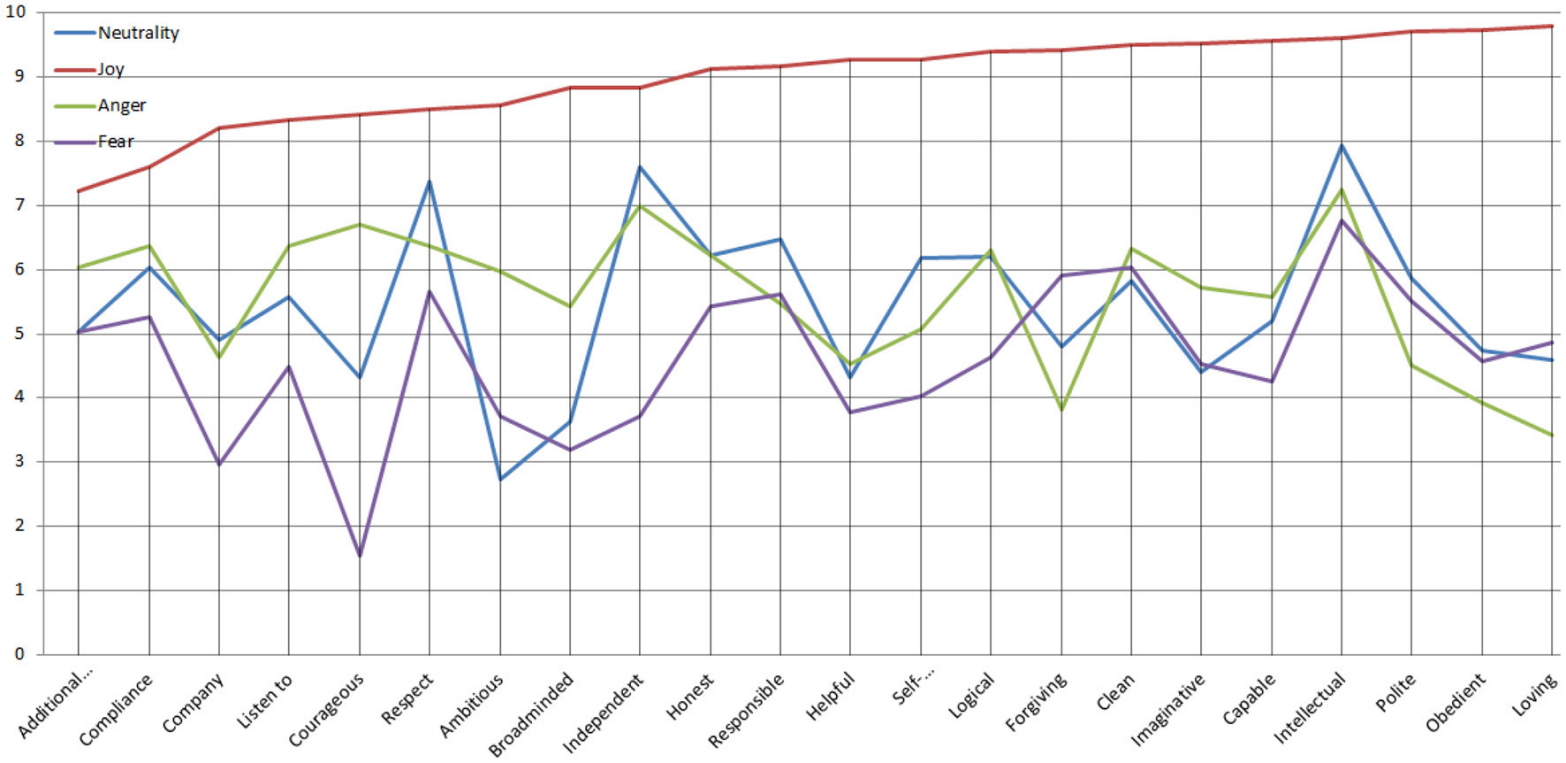
Means of children's perceptions according to the four emotional situations, showing the higher scores for joy.

Overall, the results indicated that hypothesis 1, 4 and 6 were partially confirmed, while the rest, 2, 3, and 5 were totally confirmed. The visual representation of the results is presented in Figure 2 below.

Figure 2 plots the average ratings pupils assigned to each outcome or trait (horizontal axis) on a 0–10 scale (vertical axis) for the four facial-expression conditions. The red line (Joy) sits well above the others almost everywhere, indicating that a smiling teacher consistently received the highest scores—whether for behavioral outcomes such as “Compliance” and “Listen to” or personality impressions like “Honest,” “Helpful,” and “Capable.” The green line (Neutrality) tends to occupy a middle position, while the blue (Anger) and purple (Fear) lines trail below, often dipping sharply on traits tied to warmth or cooperation (e.g., “Helpful,” “Loving”).

The graph indicates that a joyful face has a more positive impact on children's evaluations of teacher attributes and behaviors. When looking across all the significant mean differences between emotions for each trait, the largest gap appears on the *Courageous* dimension between Joy (*M* = 8.41) and Fear (*M* = 1.54), yielding a difference of 6.87 points. Joy's mean is ~5.46 times higher than Fear's. In contrast, the smallest significant gap emerges on the *Intellectual* dimension between Joy (*M* = 9.60) and Neutrality (*M* = 7.93), with a difference of 1.67 points. The smallest significant mean difference indicates that Joy's mean is about 1.21 times higher than Neutrality's.

## 6 Discussions

This study highlights the role that teachers' emotional expressions play in shaping students' perceptions, motivation, and classroom behavior. When teachers display positive emotions, particularly joy, it has a profound influence on students' willingness to participate, their respect for the teacher, and their overall enjoyment in the learning environment.

One of the most striking findings was how much more willing students were to complete exercises when the teacher expressed joy, referred to as *compliance*. This supports research by Meyer and Turner (2006), which shows that positive affect from teachers can foster greater student engagement. Interestingly, students are not just more compliant—they're eager. Joy seems to create a contagious atmosphere in the classroom where students are energized to participate. It's not just about having a happy teacher; it's about how the joy influences the entire learning climate. This emotional connection taps into intrinsic motivation, leading students to want to perform beyond basic expectations (Frenzel et al., 2009). Thus the first hypothesis is partially confirmed.

Regarding the second hypothesis, emotional displays of joy didn't just affect students' willingness to engage; it also significantly increased their *respect* for the teacher. This hypothesis is confirmed. This supports research that suggests teachers who express positive emotions are perceived as more competent and approachable (Hagenauer and Volet, 2014). Respect, in this case, seems to go beyond mere deference—it's rooted in admiration for the teacher's enthusiasm and approachability. Interestingly, this finding may link to broader social psychology literature, which shows that individuals who display positive emotions are generally perceived as more competent and trustworthy (Becker et al., 2014). In contrast, negative emotions like fear and anger not only reduce respect but can also undermine a teacher's authority, leading to classroom management difficulties.

A surprising finding was how much students reported *enjoying* the company of a joyful teacher. This goes beyond the traditional teacher-student dynamic and touches on personal rapport. The third hypothesis is confirmed. When students enjoy being around a teacher, it can lead to deeper engagement, improved classroom dynamics, and a sense of community. Pianta et al. (2012) found that strong teacher-student relationships are a key factor in academic success, particularly for students who might otherwise be disengaged. By simply being joyful, teachers can foster an emotional connection that makes students feel more at ease, which is likely to enhance learning outcomes. The fact that joy was also linked to a greater willingness to follow instructions suggests that positive emotional displays can have wide-reaching effects on student behavior.

It's not just about being liked—teachers who express joy also command greater *attention* in class. This hypothesis is confirmed. Students in the joy condition reported being more motivated to listen attentively. Emotionally supportive teachers are more successful in gaining and maintaining students' attention (Jennings and Greenberg, 2009). A joyful teacher is perceived as more enthusiastic, and this directly influences students' willingness to focus and stay engaged. In fact, emotional engagement has been found to be a precursor to cognitive engagement, meaning that students who feel emotionally connected to their teacher are also more likely to excel academically (Hamre and Pianta, 2005).

What is more, in the presence of the smiling face students are more willing to do extra exercises at home than in the neutral or fearful condition. This again indicates how a small stump can overturn a large cart, as an inexpensive smile can lead to the decision to work more at home. The hypothesis was partially confirmed. Scientific studies are in accordance with this finding. Studies showed that non-verbal immediacy, a concept referring to the use of certain clues to reduce the psychological distance between teacher and students, reinforces students' behaviors to engage in harder work (Mehrabian, 1981). A good teacher-student relationship is a reword in itself and smiling has the key role in it which mobilizes personal resources for work.

Beyond influencing immediate classroom behavior, joy also affected how students viewed their teacher's personal traits. From the 17 personality features tested, we found that joy has a significant different statistical effect than the other facial expressions in all 17 situations. Yet, I did not find significant differences in the case of students' “courageous” personality trait between joy and anger. For the personality trait “Independence,” we did not find any significant difference between joy and neutrality. Conversely, we found significant differences in all situations tested between joy and fear. Teachers who expressed joy were seen as more ambitious, broadminded, capable, and caring compared to those who displayed neutral or negative emotions. This finding complements earlier studies by Sutton and Wheatley (2003), which showed that a teacher's emotional tone shapes student perceptions of their leadership and competence. It appears that students do not just associate joy with being “nice”—they also see joyful teachers as more effective leaders. In other words, joy amplifies perceptions of both warmth and authority, a combination that can be crucial for maintaining both control and engagement in the classroom.

To deepen theoretical integration, the findings can be interpreted through the lens of Fredrickson's Broaden-and-Build Theory and Social Appraisal Theory by highlighting the mechanisms that connect emotional expressions to student responses. Smiling, as a signal of positive affect, likely activates students' parasympathetic nervous system, reducing anxiety and increasing cognitive receptiveness. This physiological shift broadens attention and promotes exploratory behaviors in learning contexts. Simultaneously, Social Appraisal Theory suggests that students interpret a smiling teacher as signaling a non-threatening, affiliative stance, which enhances motivation and engagement through perceived relational safety. These emotional cues function as regulatory signals, shaping students' academic behavior.

The large to extremely large effect sizes observed in this study suggest substantial real-world implications for classroom teaching practices. Practically, these effects mean that teachers who consistently display joyful facial expressions can significantly enhance students' compliance, respect, motivation, and positive perceptions, ultimately improving classroom climate and academic engagement. Even moderate changes in teachers' emotional expressiveness may yield noticeable improvements in student behavior and learning outcomes.

While joyful expressions have clear benefits, excessive or continuous smiling might risk diminishing perceived authenticity, potentially causing students to doubt the sincerity of the teacher. This could lead to mistrust or reduced teacher authority, suggesting that balanced emotional expressiveness is important in maintaining credibility and effective classroom management.

## 7 Conclusions

The purpose of this study was to investigate how teachers' facial emotional displays, particularly joy, fear, anger or neutrality, influence students' perceptions, motivation, and compliance in a classroom setting. The results demonstrated that positive emotional expressions, especially joy, lead to significant improvements in students' willingness to participate, their respect for the teacher, and their enjoyment of the teacher's presence. In contrast, negative emotions such as anger or fear were associated with less favorable outcomes in these areas.

This study had a few limitations. The sample size was relatively small, and all participants were drawn from a single age group, which may limit the generalizability of the findings to other student populations or age ranges. While the total sample includes 121 participants across four conditions, this size is sufficient given the large effect sizes observed (many exceeding Cohen's *d* = 0.80), which indicate strong group differences. Power analyses for ANOVA designs suggest that with medium-to-large expected effects, group sizes of ~30 provide adequate power (above 0.80) to detect significant differences. Moreover, the consistency of significant results across multiple dependent variables further supports the robustness of the findings despite the modest sample size.

Additionally, the study focused on only four emotional displays—joy, neutrality, anger, and fear—potentially missing the nuances of other emotions like sadness or surprise. Finally, the reliance on self-reported measures might introduce bias, as students' responses could have been influenced by social desirability or other factors.

Beyond the main effects of facial expressions, demographic variables such as gender and students' urban or rural background may moderate the way emotional cues are perceived and interpreted. For example, prior research indicates that girls often show greater emotional sensitivity and interpersonal attunement (Becker et al., 2014), which could make them more responsive to joyful or fearful expressions, especially in teacher-student interactions. In contrast, boys may be more reactive to expressions of authority, such as anger or neutrality, interpreting them as signals of structure or challenge. Additionally, cultural norms embedded in rural vs. urban settings may shape students' expectations of teacher demeanor—rural students might associate emotional restraint or neutrality with professionalism, whereas urban students may expect more expressiveness and relational warmth (Hargreaves, 2000; Syrjänen et al., 2018). These moderating influences suggest that the emotional impact of teachers' facial expressions may not be uniform across all students and should be further explored through interaction analyses in future research.

Further research could expand the scope of emotional displays to include a wider range of emotions and investigate how mixed emotional expressions (e.g., a combination of joy and surprise) affect student outcomes. Additionally, studies with more diverse age groups and cultural backgrounds would provide a deeper understanding of the role emotions play in different educational settings (Ahmed, 2025). The sample's geographic homogeneity—children from Suceava, Romania—limits the cultural generalizability of the findings. Emotional expression and interpretation are culturally shaped, meaning students from other regions or backgrounds might respond differently to the same facial cues. Future research should compare cross-cultural samples to examine whether these effects hold in more diverse educational contexts.

Moreover, future studies could benefit from combining self-reported data with behavioral observations to minimize bias and capture more objective measures of student engagement and compliance.

## 8 Implications of the study

The findings of this study highlight the need for teacher training programs to incorporate emotional intelligence development alongside pedagogical skills, as the ability to express positive emotions like joy significantly enhances student engagement, motivation, and respect. In today's classrooms, where distractions and mental health challenges are on the rise, teachers who can manage and project positive emotions can create more effective learning environments. Moreover, with the increased use of digital learning and fragmented attention spans, cultivating a positive emotional climate is more crucial than ever for improving academic outcomes (Rad et al., 2023; Runcan et al., 2023) and promote child resilience (Marici, 2015a). Implementing strategies to support teachers in maintaining positive emotions—even in high-stress environments—can help combat classroom disengagement and reduce behavioral issues. Finally, educational policymakers should recognize the impact of teacher emotions on student success and consider integrating emotional wellbeing into teaching standards (Turliuc and Marici, 2013), providing emotional support systems for teachers to ensure they can foster productive and engaging classrooms (Marici, 2015b,c).

An innovative approach to emotion-based teacher training would involve integrating real-time biofeedback and emotion recognition technologies, allowing teachers to observe how their facial expressions are perceived by students. These tools can be embedded into training simulations that replicate classroom dynamics, both in-person and online. Policy recommendations should support the development of national standards for emotional literacy in teaching, ensuring it becomes a core competency. In virtual classrooms, practical implementation could include interface designs that prompt emotional check-ins or provide live cues to maintain facial expressiveness, ensuring relational presence even through screens.

## Data Availability

The datasets presented in this study can be found in online repositories. This data can be found here: https://doi.org/10.5281/zenodo.16740175.
